# Perioperative Ketamine and Cancer Recurrence: A Comprehensive Review

**DOI:** 10.3390/jcm13071920

**Published:** 2024-03-26

**Authors:** Juan Alberto Rodriguez Arango, Tamara Zec, Maher Khalife

**Affiliations:** Department of Anaesthesiology, Institut Jules Bordet, Université Libre de Bruxelles, 1070 Bruxelles, Belgium

**Keywords:** cancer, postoperative recurrence, ketamine, s-ketamine, anesthesia, inflammation, microbiota, biomarkers, drug response

## Abstract

Cancer is a significant global health threat and a leading cause of death worldwide. Effective early-stage interventions, particularly surgery, can potentially cure many solid tumors. However, the risk of postoperative cancer recurrence remains high. Recent research highlights the influence of perioperative anesthetic and analgesic choices on the fate of residual cancer cells, potentially affecting recurrence risks. Among these agents, ketamine—a well-known anesthetic and analgesic—has garnered interest due to its antitumor properties, mainly through inhibiting the N-methyl-D-aspartate (NMDA) receptor found in various cancer tissues. Additionally, ketamine’s potential immunomodulatory effects, given the expression of NMDA receptors on immune cells, suggest that it plays a significant role during the perioperative period. This review synthesizes current evidence on ketamine’s impact on cancer cell biology, inflammation, immune modulation, and the role of the gut microbiota, proposing ketamine as a promising agent for enhancing oncological outcomes.

## 1. Introduction

Ketamine, an N-methyl-D-aspartate (NMDA) antagonist, is a key component in multimodal analgesia strategies and serves as an anesthetic agent for procedural sedation and anesthesia induction in patients with hemodynamic instability [1]. Although initially synthesized in 1962, recent decades have seen a resurgence in its use, driven by a renewed examination of its pharmacologic properties [1,2]. Recent studies suggest its potential application in oncology, where it functions as an immunomodulator, attenuating the inflammatory response induced by surgery. Utilizing this mechanism, ketamine may help to mitigate metastasis and cancer progression during the perioperative period.

GLOBOCAN 2020 identified cancer as a principal cause of death globally, estimating 19.1 million new cases and recording nearly 10 million deaths in 2020 [3]. Notably, metastasis accounts for up to 90% of these deaths [4,5]. The projection for new cancer cases is alarming; new cases are anticipated to reach 24 million by 2035 [6]. As surgery remains a primary curative treatment, many cancer patients will undergo at least one surgical procedure [7]. Hence, the responsibility of the multidisciplinary medical team is to ensure optimal care in order to prevent cancer recurrence and progression.

Numerous studies have been conducted to determine the effects of various anesthetic and analgesic techniques on cancer cells, both in vitro and in oncological patients. These techniques have been meticulously assessed regarding their possible impact on immune and inflammatory responses in the perioperative period, as well as their potential implications for outcomes such as metastasis and recurrence [7,8,9,10]. Within this scope, this article aims to offer an exhaustive review of current insights into ketamine and s-ketamine’s impact on inflammation, immunomodulation, and cancer cell biology.

## 2. Methods

The most recent and relevant evidence was collected from different databases, including Medline, Google Scholar, and Cochrane library. The following search terms were used to conduct the literature search: ketamine, s-ketamine, cancer, cancer recurrence, anesthesia, inflammation, immune function, metastasis. Regarding the article type, there were no restrictions as long as articles were written in English. To locate more potentially relevant studies, a manual search was conducted of all retrieved articles and pertinent reviews. The authors determined the suitability for inclusion based on a broad and impartial selection of pertinent and up-to-date research.

## 3. Postoperative Cancer Recurrence

The mechanisms underlying post-surgical cancer recurrence following primary tumor resection remain elusive. Several factors during the perioperative period contribute to this phenomenon. These include local recurrence due to the proliferation of residual cells at the tumor resection site, lymph node metastasis, distant organ metastasis from circulating tumor cell seeding, and seeding within body cavities [5]. Moreover, surgical stress and tumor manipulation can result in pathophysiological changes in the cancer cell microenvironment. Such changes can incite inflammation, tissue hypoxia, angiogenesis, and immunosuppression [8]. This microenvironment encompasses non-cancerous stromal cells, immune cells, the extracellular matrix, chemokines, cytokines, and numerous other elements [11]. Perturbations in this environment can occur due to the activation of the sympathetic nervous system, the hypothalamic–pituitary–adrenal axis, elevated circulating inflammatory mediators, hypothermia, allogenic blood transfusion, and potentially specific anesthetic or analgesic agents [7,8,12,13].

## 4. Properties of Ketamine and Clinical Uses

Ketamine, an NMDA antagonist, possesses profound anesthetic and analgesic properties both centrally and peripherally [1,14]. Its analgesic properties, particularly at subanesthetic doses—defined as ≤0.5 mg/kg IV bolus or an infusion rate of <0.5 mg/kg/h, resulting in plasma concentrations between 100 and 200 ng/mL—present a promising avenue for perioperative pain relief, potentially reducing the reliance on opioids [15,16].

The acute analgesic effects of ketamine are generally attributed to the blockade of the phencyclidine binding site of NMDA receptors (NMDR) on nociceptive neurons [14]. However, ketamine also interacts with various receptor systems, including opioid, monoamine, cholinergic, adrenergic, purinergic, adenosine, AMPA, kainate, and GABA, while also inhibiting serotonin, dopamine reuptake, and certain voltage-gated channels [14,17,18,19,20,21]. The emergence of s-ketamine or esketamine, the dextrorotary enantiomer of ketamine, has further enhanced the compound’s profile by offering a version with enhanced analgesia and diminished side effects [22]. It is worth noting that, at anesthetic doses (≥1.0 mg/kg IV), ketamine can induce psychotomimetic effects and heightened sympathetic stimulation, potentially disrupting the immunomodulatory process [2,23].

The shift towards ketamine and its enantiomers in multimodal analgesia is supported by comprehensive reviews and meta-analyses, spotlighting its efficacy in alleviating postoperative pain and minimizing opioid use [15,24]. A recent meta-analysis by Wang et al. [25] advocates for subanesthetic doses of s-ketamine, indicating its capacity to reduce postoperative pain intensity and opioid consumption. Showing significant improvements in resting pain scores at 4, 12, and 24 h with s-ketamine versus placebo (*p* < 0.00001; *p* = 0.001; *p* = 0.02, respectively). At 4 and 12 h after surgery, the consumption of morphine was also significantly reduced in the s-ketamine group. Other meta-analyses investigating ketamine have yielded consistent outcomes, as highlighted by a comprehensive Cochrane review spanning 130 studies and 8341 participants [26,27,28]. These collective findings substantiate the potential therapeutic efficacy of ketamine in improving postoperative pain management.

Low doses of ketamine have been shown to protect against neurotoxicity from inflammatory pain [29]. However, evidence indicates that ketamine can induce neuronal apoptosis in a dose-dependent manner via reactive-oxygen-species-mediated mitochondrial pathways [30,31]. Ketamine’s role as an adjunct in postoperative pain management is undeniable; however, its utilization requires a careful balance to maximize its therapeutic potential while minimizing adverse effects. These effects can include sympathomimetic stimulation, CNS excitation, oversedation, and visual disturbances or hallucinations [2,32]. Fortunately, the adverse outcomes that necessitate discontinuing the drug in the perioperative period are uncommon and self-limited [32].

Recently, ketamine has gathered significant attention due to its inherent anti-inflammatory and immunomodulatory properties, as well as its direct effects on cancer cell biology, which have yet to be fully elucidated [14,23]. The presence of NMDARs in lymphocytes and natural killer cells implies their potential significance in perioperative immunomodulation [33]. Earlier research indicates antitumor effects through NMDAR inhibition in various cancer cell types [34,35,36,37]. Based on these data, we contend that ketamine presents promising benefits for the oncological population.

## 5. Anti-Inflammatory Effect of Ketamine

Elevated levels of proinflammatory cytokines, such as interleukin 6 (IL-6) and tumor necrosis factor alpha (TNF-α), are known to enhance cancer cell proliferation and survival. This enhancement occurs through the inhibition of the antitumor functions of effector cells, including natural killer (NK) cells, CD4+ T helper type-1 cells, and CD8+ cytotoxic lymphocytes [38]. Elevated IL-6 plasma/serum concentrations are associated with various postoperative complications [39,40,41,42], ranging from major cardiopulmonary complications after general thoracic surgery to heightened risk of coronary heart diseases [43], postoperative morbidity after cardiac surgery [44,45,46], and cognitive dysfunction following coronary artery surgery [47]. Recent data indicate that the perioperative overproduction of inflammatory cytokines is significantly associated with cancer progression and recurrence in almost all major types of cancer [48,49].

Ketamine displays a broad spectrum of anti-inflammatory effects, suggesting its participation in the initial stages of the inflammatory cascade [50]. The proposed mediation of ketamine’s anti-inflammatory effect is linked to the reduction in microglia activation, evident through the inhibition of extracellular-signal-regulated kinase 1/2 phosphorylation in primary cultured microglia [51], or the suppression of large-conductance calcium-activated potassium channels in microglia [52]. Blocking these channels curtails the transmission of peripheral inflammatory signals that activate microglia, leading to a reduction in pro-inflammatory cytokine synthesis in the central nervous system’s immune cells [50].

A key mechanism of ketamine’s action is its suppression of nuclear factor-κB (NF-κB), an intracellular protein essential for initiating the transcription of cytokine-coding genes in immune cells [50,53]. Additional hypothesized mechanisms include the inhibition of proinflammatory cytokine production (IL-6 and TNF) [54,55,56], the suppression of neutrophil functions [57], adenosine release [58], the reduction in nitric oxide synthesis in macrophages [59], and the activation of the Wnt5a signaling pathways [50]. Whether ketamine and local anesthetics employ a shared anti-inflammatory mechanism remains debatable. While ketamine diminishes neutrophil activation, it retains endothelial cytokine production, thus supporting an anti-inflammatory response without impacting local healing processes [14,60,61].

Additionally, ketamine stimulates the heme oxygenase enzyme system, which, during heme catabolism, generates metabolites (biliverdin, free iron, and CO) renowned for their robust protective properties against sepsis and ischemia/reperfusion injuries in laboratory studies [50]. Further research is required to elucidate the exact contributions of these mechanisms to ketamine’s protective anti-inflammatory effects.

### 5.1. Laboratory Studies

Ketamine has been observed to predominantly influence the local inflammatory process during its early stages. Its regulatory impact becomes more pronounced when administered preemptively, prior to any inflammatory stimulus [50]. Numerous in vitro and in vivo studies have highlighted ketamine’s ability to substantially reduce proinflammatory cytokine concentrations, while apparently not affecting the production of anti-inflammatory cytokines [50]. In vitro examinations involving human whole blood exposed to lipopolysaccharides (LPS) and a spectrum of ketamine concentrations (ranging from 20 to 100 mcg/mL) have unveiled a discernible decline in pro-inflammatory cytokines such as IL-6, IL-8, IL-1 beta, and TNF-α [62,63,64]. Corroboratively, in vivo assessments using murine models documented a notable reduction in IL-6 and TNF-α concentrations, along with a rise in survival across diverse conditions [54,65,66,67,68] (Table 1).

Ketamine may interfere with early detection mechanisms, including Toll-like receptors (TLRs), as suggested by experimental studies using an endotoxic shock rodent model [50]. The interaction of ketamine with TLR-derived mechanisms is particularly intriguing, given that most in vitro anti-inflammatory properties of ketamine have been demonstrated using LPS, which are specific agonists of TLRs type 4 and major constituents of gram-negative bacteria [50]. The exact relationship between ketamine’s interaction with TLR-derived mechanisms and its antagonism in the NMDAR system remains to be determined [50]. However, it is unmistakably evident that ketamine plays a central role in the inflammatory cascade, exerting its influence both locally and systemically through a confluence of mechanisms. However, more exhaustive clinical studies are essential to validate the potential oncological advantages of ketamine.

### 5.2. Clinical Studies

Ketamine’s integration into pain management presents a dual opportunity: the enhancement of analgesia and mitigation of pain mediator synthesis and release. Uncontrolled pain, or even suboptimally managed pain, is recognized to elevate proinflammatory cytokine levels [75].

An empirical validation of ketamine’s proficiency in dampening the IL-6 response post-cardiopulmonary bypass (CPB) was achieved. Specifically, the infusion of a minor ketamine dosage (0.25 mg/kg IV bolus) in tandem with fentanyl anesthesia was observed to moderate the serum IL-6 response across the initial 7 days succeeding CPB without perturbing hemodynamic equilibrium during the various phases of surgery [76].

The data mirror the study led by Beilin et al. [77], wherein 36 patients scheduled for abdominal surgeries were split into two arms. Introducing minor ketamine doses (0.15 mg/kg as a single IV bolus) before the induction of anesthesia resulted in the dampening of proinflammatory cytokine (IL-6 and TNF-α) release. Notably, IL-6 increased in the control but remained stable in the ketamine group 4 h post-surgery, presenting a statistically significant difference (*p* < 0.05). However, this difference was transient; both cohorts evidenced heightened IL-6 levels 24, 48, and 72 h post-procedure.

A systematic review and meta-analysis conducted by Dale et al. [14] advocated for intraoperative ketamine’s role in various surgeries, accentuating its discernible modulatory effect on the early postoperative IL-6 response. This conclusion was derived from the synthesis of 14 research studies involving 684 patients. Nevertheless, a limitation remains: the primary focus of these studies was on IL-6 modulation, rather than a comprehensive assessment of patient outcomes.

These findings suggest that, in surgical settings, ketamine may effectively suppress early postoperative IL-6 inflammation. Nevertheless, there is a need for further research to clarify the clinical implications of this suppression, particularly in the context of oncological surgeries. Future studies should focus on a wider range of clinical outcomes, thereby enhancing our understanding of ketamine’s influence in the perioperative period.

More recent studies have shown similar results regarding ketamine’s anti-inflammatory effect. In a randomized clinical trial involving 104 patients undergoing colorectal surgery, different sub-anesthetic doses of ketamine were analyzed. The study found that a single 0.3 mg/kg dose effectively reduced the serum levels of IL-6, IL-8, and TNF-α, and improved postoperative emotional responses [78]. Another prospective observational analysis investigated low-dose ketamine’s impact on inflammatory biomarkers following off-pump coronary artery bypass grafting. The research, involving 60 patients, highlighted that a low 0.5 mg/kg dose of ketamine significantly reduced the postoperative levels of C-reactive protein (CRP) and IL-6 (*p* < 0.001) up to 48 h after surgery [79].

In the context of radical prostatectomy, a randomized controlled trial focused on the effects of single versus repeated doses of s-ketamine on pro-inflammatory cytokines; s-ketamine was injected as a single intravenous dose of 0.5 mg/kg or repeated doses of 0.2 mg/kg were injected at 20 min intervals until 30 min before the end of surgery. The study revealed that s-ketamine significantly decreased TNF-α and IL-6 compared to the control group (*p* < 0.001) up to 4 h after surgery, especially when given in repeated doses [80].

Another randomized clinical trial assessed ketamine’s effects when used as an adjuvant to bupivacaine for local wound infiltration in patients undergoing abdominal hysterectomy. The findings revealed a significant attenuation in inflammatory cytokines at 6 and 24 h postoperatively, IL-6 (*p* < 0.028 and *p* < 0.013 respectively), and TNF-α (*p* < 0.011, *p* < 0.002 respectively), an increase in IL-10, stable IL-1β levels, and a reduction in postoperative morphine consumption [81].

Furthermore, in the context of major depressive disorder, a study involving 60 patients explored ketamine’s role in modulating peripheral inflammatory cytokines [82]. Patients received six 0.5 mg/kg ketamine infusions over 12 days, leading to a noteworthy downregulation in a broad spectrum of cytokines, correlating with symptom improvement [82]. This study not only reinforced ketamine’s anti-inflammatory properties but also its potential as a novel treatment for depressive symptoms, possibly mediated through inflammatory pathways.

However, not all analyses sing in harmony. A contemporaneous meta-analysis unveiled contrasting insights regarding ketamine’s effects on postoperative systemic inflammation in surgical patients. While subanesthetic ketamine doses did not notably reduce IL-6 levels, they did produce a remarkable downturn in CRP levels 24 h post-surgery, suggesting potential modulation in the early postoperative inflammatory milieu [83].

Caution should be exercised in interpreting these findings. The meta-analysis was punctuated by significant variability among studies and was generally skewed towards lower quality [83]. This underscores the need for more sophisticated, well-calibrated studies, especially as major surgeries naturally invoke robust inflammatory reactions—contexts where ketamine might emerge as a decisive factor.

## 6. Immunomodulatory Effect of Ketamine

Post-surgical immunosuppression is an inherent challenge, particularly within oncology, due to its possible implications for outcomes such as tumor recurrence, metastasis, and accelerated cancer progression [84,85]. The critical roles of NK cells and CD8+ T cells in tumor surveillance cannot be overstated; their direct cytotoxicity toward malignancies is a fundamental aspect of the body’s defense against cancer proliferation and metastatic activity [7,86,87].

Opiate-heavy anesthesia is facing increased scrutiny, particularly regarding its potential to exacerbate the dampening of NK cell activity triggered by surgery and to reduce the synthesis of IL-2 and interferon-gamma (INF-γ)—two crucial cytokines that drive T-cell proliferation and regulatory functions. This has led to concerns regarding the use of opiate-based anesthetic strategies, especially in cancer patients, due to the possible prolonged immunosuppressive effects [71,74].

The imperative for novel therapeutic approaches, particularly those customized to the intricate demands of oncological patient care, cannot be overstated. Administering ketamine in subanesthetic dosages could introduce a multitude of advantages for patients with cancer, attributable to its robust immunomodulatory properties. Through the potential reduction in postoperative metastasis and tumor recurrence risks, ketamine appears well-positioned to fill a significant clinical gap.

The growing body of evidence highlighting ketamine’s immunomodulatory effects in oncological contexts is compelling, but there is still much to uncover. A thorough and detailed investigation is crucial to understand the complex interactions of ketamine’s impact on immune responses, particularly in tumor biology. Rigorous research, guided by well-designed clinical trials, is essential. There are two primary objectives: to obtain a comprehensive understanding of ketamine’s immunological implications and to evaluate its effectiveness in reducing postoperative cancer recurrence and metastasis. Only through such systematic exploration can we fully harness ketamine’s potential as an immunomodulatory agent in surgical oncology.

### 6.1. Laboratory Studies

The effects of ketamine on immunomodulation remain a topic of debate, with some evidence pointing to potentially adverse outcomes associated with higher doses. The activation of the NMDAR in lymphocytes induces an excitotoxic reaction, suggesting that human lymphocyte activity may be regulated by neuroendocrine control via glutamate receptors similar to those in neuronal cells [33]. Melamed et al. [88] showed that, in murine models, ketamine (injected intraperitoneally at 80 mg/kg) increased MADB106-induced lung metastases over 2.5 times and notably suppressed NK activity post 74 mg/kg IV injection when compared to a control group. Another study inferred that this effect arises from ketamine’s beta-adrenergic stimulation [88]. Ketamine is known to interact with alpha-1 and beta-2 adrenoreceptors and inhibit catecholamine reuptake [20]. Adrenergic stimulation may suppress NK activity [70,88].

Zhou et al. [74] investigated the suppressive effects of ketamine and morphine on activated T lymphocytes in patients suffering from refractory cancer pain. Their results indicated that both drugs exerted a dose-dependent suppression of IL-2 and INF-γ levels in vitro. However, the suppressive effect of low-dose ketamine (100 ng/mL) appeared minimal. A subsequent in vitro study by the same team affirmed these results when assessing the effects of morphine and low-dose ketamine (100 ng/mL for 24 h) on T cells from patients with refractory cancer pain [89].

In their subsequent investigation, Zhou et al. [89] found that treatment with morphine, both solo and in combination with ketamine, inhibited immune functions in patients. This was reflected by decreases in CD4+ and CD8+ T cell counts, CD4+/CD8+ ratios, the concentrations of IL-2 and INF-γ in the supernatant, the mRNA expression levels of IL-2 and INF-γ, and the activation of NF-κB. However, there was no marked difference between the combined morphine and ketamine treatment and morphine-only treatment in terms of immunosuppressive effects [89]. These findings further emphasize the notion that low-dose ketamine may not induce immunosuppression, but would instead modulate the immune response in patients with refractory cancer pain.

Forget et al. [10] conducted animal studies on the impact of ketamine on NK cell activity post-surgery. They discovered that administering ketamine at a 10 mg/kg dose intraperitoneally significantly dampened NK cell activity (5.4 ± 1.0 vs. 15.6 ± 2.9%, *p* < 0.05) in nonoperated male Wistar rats. Nonetheless, post-surgery, the NK cell activity mirrored that seen in saline-treated animals. Interestingly, ketamine enhanced NK activity eight days post-surgery compared to the control group of nonoperated animals treated with ketamine.

This research team also determined that, upon inducing lung metastasis in murine models using a chosen variant of mammary adenocarcinoma MADB-106, ketamine notably decreased the count of lung metastases relative to the control group (200 ± 17 vs. 108 ± 10, *p* < 0.05) [10].

There is a pressing need for more research to thoroughly understand the mechanisms behind these effects and their clinical ramifications. Comprehensive studies, spanning in vivo experiments and clinical trials, are crucial to identify the best dosages and therapeutic techniques, balancing the potential immunomodulatory benefits of ketamine against its possible adverse outcomes in oncologic patients.

### 6.2. Clinical Studies

It has been observed that low perioperative NK activity levels can be associated with increased morbidity and mortality from cancer, particularly in cases of colorectal, breast, lung, and head and neck malignancies [87,90]. In a prospective, open-label, cross-sectional study by Jobin et al. [91] involving 872 high-risk participants screened for colorectal cancer (CRC) using colonoscopy, it was deduced that individuals with diminished NK cell activity (median level of 86.0 pg IFNG/mL vs. 298.1 pg IFNG/mL, *p* = 0.0002) had a risk of CRC that was ten times greater than those with heightened NK cell activity (95% CI, 3.03–34.9).

Ketamine can counteract alterations in immune function stemming from anesthesia and surgical procedures [77] (Table 2). One randomized controlled trial determined that a single subanesthetic bolus of ketamine (0.15 mg/kg IV) given five minutes before the induction of general anesthesia for elective abdominal surgeries resulted in a marked reduction in proinflammatory cytokines four hours post-surgery. Notably, IL-2 was preserved at preoperative levels, and no significant differences in natural killer cell cytotoxicity cells (NKCC) were observed compared to the control group [77].

Conversely, Cho et al. [23], in their prospective randomized controlled trial, inferred that intraoperative subanesthetic low-dose ketamine (IV bolus of 0.25 mg/kg administered five minutes before surgical incision, followed by a 0.05 mg/kg/h infusion until surgery conclusion), when used as an adjunct to desflurane, did not reveal any substantial benefits to postoperative NK cell activity in patients undergoing colorectal cancer surgery. There was a significant reduction in NK cell activity after the operation in both groups, but the shift was not significantly different between the groups in the linear mixed-model analysis (*p* = 0.47). Alterations in the levels of IL-6, TNF-α, CRP, and carcinoembryonic antigen did not differ significantly between the groups (*p* values of 0.27, 0.69, 0.99, and 0.97, respectively). Cancer recurrence rates within two years of surgery remained comparable between groups (10% vs. 8%, *p* = 0.62) [23].

Nevertheless, the limitations of this study must be acknowledged, particularly concerning the complex interactions between various anesthetic regimens and immune function. Previous research has shown that both volatile anesthetics and opioids can impair NK cell activity, potentially leading to post-surgical immunosuppression [71,93,94].

A recent randomized controlled trial assessed the impact of three distinct doses of s-ketamine (0.25, 0.5, and 0.75 mg/kg at induction, followed by infusions of 0.25, 0.5, and 0.75 mg/kg/h, respectively) on immune-inflammatory function in patients undergoing modified radical mastectomy [92]. The data indicated that s-ketamine doses of 0.5 mg/kg and 0.75 mg/kg had a diminished impact on patients’ cell-mediated immune function, resulting in a more attenuated inflammatory response. The study also revealed that the percentage and absolute counts of CD3+ and CD4+ cells in the s-ketamine groups exceeded those in the control group at specified postoperative intervals. In comparison to the control group, the concentrations of white blood cells, neutrophils, hypersensitive C-reactive protein, the neutrophil-to-lymphocyte ratio, systemic inflammation response index, and the systemic immune-inflammation index at the conclusion of surgery and 24 h postoperatively in the three s-ketamine dose groups were notably lower, while lymphocyte counts were significantly elevated. No marked difference in the percentage and absolute counts of NK cells and B lymphocytes was observed among the four groups [92]. Notwithstanding this, the trial had certain limitations. The limited sample size could potentially overstate the treatment effect. To comprehensively validate the results herein, follow-up investigations utilizing larger sample sizes and multicenter studies are imperative. Furthermore, the study did not conclusively determine the optimal dose.

The existing evidence presents a varied picture; there is an evident lack of dosage standardization across studies, and there is a shortage of clinical trials that could be used to draw definitive conclusions. However, low-dose ketamine or s-ketamine seem to be a safe intervention that may not trigger clinically significant immunosuppression. In fact, it might beneficially adjust the immune response. However, it is essential to factor in the entire anesthetic regimen to accurately measure this outcome. More randomized controlled trials are indispensable.

## 7. Direct Effect of NMDA Antagonism on Cancer Cell Biology

Functional NMDARs are expressed in various cancer cells [34]. This has been confirmed across several tumor cell lines, such as neuronal tumors (astrocytoma, glioma, and neuroblastoma), rhabdomyosarcoma, medulloblastoma, thyroid carcinoma, lung carcinoma, colon carcinoma, breast carcinoma, T-cell chronic lymphocytic leukemia, multiple myeloma [34], laryngeal carcinoma [95], pancreatic carcinoma cells [35], and prostate carcinoma [96].

While the role of the NMDAR in oncogenic behavior remains enigmatic, it may be pivotal to tumor development, growth, and metastasis [97]. Agonizing the NMDAR boosts intracellular calcium levels, activating Ca^2+^-dependent systolic guanylate cyclase [21]. This influx of calcium into the cytoplasm results in the activation of secondary messengers, which can then engage various proteins, including transcription factors that govern tumor cell activities, behavior, and progression [98].

Calcium acts as a primary messenger in multiple signaling pathways governing tumor development, specifically impacting cancer cell proliferation and death [99]. The activation of the NMDAR has been proven to escalate calcium levels and amplify the function of Ca^2+^/calmodulin-dependent protein kinase II (CaMK II), fostering cancer cell migration and promoting glycolysis [100]. Concurrently, the misregulation of c-Myc expression appears across various cancer types. This oncogenic transcription factor plays central roles in myriad cancer-related processes, particularly glycolysis [100].

Proteins interacting with NMDAR and subsequent signaling pathways exhibit shared characteristics between neuronal processes and metastatic cancer mechanisms, including aspects like cell adhesion, migration, and survival [101]. This convergence implies that insights from the neuronal domain could reveal the signaling and structural associates of NMDAR, highlighting potential therapeutic targets in oncology [101].

The potential therapeutic implications of understanding the NMDA receptor’s influence on the oncogenic behavior of cancer cells are vast. Both preclinical and clinical research initiatives are warranted to clarify the exact mechanisms by which the NMDAR impacts cancer progression. Exploring potential therapeutic interventions targeting the NMDAR could lead to significant advancements in cancer treatment.

### Laboratory Studies

The NMDA receptor’s influence on cancer cell oncogenic behavior remains a tantalizing but underexplored dimension of cancer research. The potential antitumor effects of NMDAR antagonists, notably ketamine and s-ketamine, have been explored in various cancer cell types [35,36,37]. These studies highlight the growing potential of NMDA antagonism as a therapeutic pathway in oncology (Table 3).

While inhibiting cancer cell proliferation may postpone tumorigenesis, it seldom achieves total tumor eradication. In contrast, inducing cancer cell apoptosis presents a profound therapeutic target in oncology, with the potential to diminish or even obliterate tumors [36]. Zhou et al. scrutinized the apoptotic impacts of ketamine on lung adenocarcinoma cells in vitro. By exposing these cells to an array of ketamine concentrations (0, 1, 10, and 100 μmol/L) for 24 h, they discerned a marked induction of apoptosis, attributed to the upregulation of CD69 expression—a known marker of activated cells, like lymphocytes and NK cells [36]. The apoptosis driven by CD69 may be attributed to its association with an N-terminal fragment of calreticulin on the cell surface, which is potentially involved in the CD69-mediated mechanism of ketamine-induced apoptosis [36].

Ketamine, when employed as an antagonist of NMDARs in colorectal cancer cells, diminishes the expression of vascular endothelial growth factor (VEGF), hypoxia-inducible factor 1-alpha (HIF-1α), phosphorylated AKT, phosphorylated extracellular signal-regulated kinase 1/2 (ERK), and phosphorylated CaMK II. It also reduces the intracellular Ca^2+^ level in a concentration-dependent manner (1, 5, 10 μg/mL). This leads to the inhibition of VEGF expression and cell migration in colorectal cancer cells in vitro [37].

Moreover, ketamine curbs the phosphorylation of ERK and AKT, resulting in the suppression of HIF-1α expression [37]. Existing evidence underscores HIF-1α’s role in stimulating angiogenesis and migration through the activation of downstream genes [106,107]. Elevated HIF-1α levels in cancerous tissues have been linked to adverse outcomes in oncology patients, suggesting their potential as a therapeutic target [37].

Elevated HIF-1α levels in cancerous tissues have been linked to adverse outcomes in oncology patients, suggesting their potential as a therapeutic target [100]. With NMDAR activation amplifying CaMK II activity—which is integral to the regulation of cancer cell proliferation, differentiation, and survival—and the aberrant c-Myc functioning as a key oncogenic transcription factor that is vital for glycolysis and diverse cancer processes [100], the implications are evident. In vitro testing involved treating colon cancer cells with various ketamine concentrations (1, 5, and 10 μg/mL) for 4 h. Concurrent in vivo testing saw ketamine (5 mg/mL) being administered to murine CT26 colon cancer cells, which were subsequently grafted onto BALB/c mice [100]. Ketamine successfully curtailed the mRNA expression levels of primary glycolysis enzymes, mitigating the glycolysis capacity in colon cancer cells, which hampers cancer cell viability and migration [100]. To truly understand the benefits of blocking the NMDAR in colon cancer patients, comprehensive translational research is essential, encompassing an analysis of resultant clinical outcomes.

He et al. revealed that ketamine hampers liver cancer cell viability and triggers ferroptosis both in vitro and in vivo [102]. For the in vitro component, human liver cancer cell lines HepG2 and Huh7 were exposed to a 10 μg/mL concentration of ketamine. The in vivo segment involved treating BALB/c mice with ketamine (20 mg/kg) intraperitoneally post-tumor implantation. Ferroptosis, a unique, regulated form of cell death, diverges from conventional cell apoptosis [102] and is typified by an uptick in intracellular reactive oxygen species (ROS) production, leading to oxidative cell death [108]. The post-ketamine-treatment accumulation of Fe^2+ iron, lipid ROS, and malondialdehyde (MDA), coupled with a decline in glutathione peroxidase 4 (GPX4) levels, points towards lipid peroxidation, possibly channeled through the lncPVT1/miR- 214-3p/GPX4 regulatory axis [102].

However, it is essential to acknowledge contrasting findings. A specific in vitro study demonstrated that ketamine could amplify the invasion and proliferation of the MDA-MB-231 breast cancer cell line by fostering antiapoptotic effects, primarily through Bcl-2 upregulation post a 24 h ketamine treatment at 100 μM [104]. This suggests that not all cancer cell types would benefit from ketamine treatment.

Chen et al. [103] also found that, in a ketamine addiction model using C57BL/6JNarl mice, ketamine significantly bolstered breast tumor growth. This surge was observed alongside an increased level of miR-27b-3p, human epidermal growth factor receptor 2 (HER2), and epidermal growth factor receptor (EGFR). In this model, mice received daily intraperitoneal injections of ketamine (30 mg/kg) for 14 days. Following this regimen, EO771 cells were introduced to develop a triple-negative breast cancer tumor model [103]. The takeaway is that chronic ketamine use might inadvertently aid in breast tumor progression.

An in vitro experiment demonstrated that two types of pancreatic carcinoma cells, PaTu8988t and Panc-1, express the NMDAR subtype R2a [35]. Notably, high-dose (1000 μM) treatments with ketamine, s-ketamine, and MK 801 substantially inhibited cell proliferation across all tested lines, including pancreatic cancer cells, inducing apoptosis in the process [35]. This anti-proliferative effect potentially arises from immune function suppression, which occurs at high doses.

In a separate investigation on the role of NFATc2—a specific NFAT protein implicated in carcinogenesis through its interaction with various transcription factors [109]—in pancreatic cancer, it was revealed that treatments with 5 μM of ketamine and s-ketamine initially decreased the levels of NFAT transcription factors in the nuclei of PaTu8988t cells at the 24 and 48 h marks [105]. However, a significant resurgence in NFATc2 expression was observed after 72 h of stimulation [105], highlighting the potential for adverse effects following prolonged exposure.

Despite the presence of conflicting evidence, the data reviewed here advocate for a promising strategy in cancer treatment via the targeted blockade of unique oncogenic pathways through NMDA inhibition. However, further research is essential to fully unravel the underlying mechanisms and to refine the therapeutic potential of ketamine in cancer therapy.

Future studies should prioritize examining the effects of ketamine on solid tumors in vivo, coupled with an exploration of its potential clinical implications. Ketamine, at a reduced concentration of 10 μmol/L, has already showcased significant potential as a novel anti-cancer agent [36]. Ensuring the success of upcoming research necessitates adherence to standardized protocols that mirror real-world clinical settings, incorporating low dosage evaluations and appropriate treatment durations.

As our understanding of the NMDAR in cancer cells expands, it is becoming clear that this complex pathway warrants an in-depth exploration. Determining the specifics of when and how the NMDA receptor’s modulation might either aid or impede cancer progression could yield promising therapeutic prospects.

## 8. Role of the Gut Microbiota

Recent research has highlighted the importance of changes in gut microbiota, underscoring its links with pain, inflammation, immunomodulation, and disruptions in the epithelial barrier [110,111,112]. Such microbial imbalances, compounded by immune dysregulation, may predispose individuals to the onset and escalation of neurological conditions [111,113], and even the progression of cancer [114,115]. Notably, the reduction in gastrointestinal motility and the incidence of opioid-induced constipation can amplify these adverse effects [111]. Ketamine has been observed to potentially reduce the number of bacteria associated with inflammatory processes in the gut and restore bacteria with anti-inflammatory properties, as evidenced by rodent laboratory studies [116]. However, these findings are preliminary and warrant cautious interpretation. Further research is imperative to validate these effects of low-dose ketamine in a clinical context.

Investigating the precise ways in which ketamine and anesthesia techniques influence the gut microbiota’s composition is essential. Furthermore, understanding the repercussions of this dysbiosis for postoperative results, especially after oncological surgeries, is indispensable. Exploring multimodal analgesia strategies to reduce opioid consumption in the perioperative period might present a means to counter potential adverse ramifications.

## 9. Discussion

The perioperative phase is identified as a pivotal juncture for the potential spread and metastasis of cancer [7]. A combination of factors—including the surgical dissemination of cancerous cells into the bloodstream, the increase in growth factor concentrations during wound healing, and perioperative immunosuppressive episodes that weaken anticancer immune vigilance—are instrumental in this process [7,117]. We urge our readers and fellow researchers to persistently scrutinize this intriguing compound for its latent potential. Determining its exact anti-inflammatory and immunomodulatory impacts post major oncological and non-oncological surgeries, as well as comprehending the significance of NMDA antagonism in cancer treatments, is vital. Although subanesthetic dosages are deemed safe and tend to curtail undesired side effects, there is a pressing need for standardization in research methodologies. This facilitates the recommendation for an apt timing and dosing protocol. Comprehensive translational research, including the initiation of randomized controlled trials, is essential for informed clinical decision-making during the perioperative period of oncological patients. Additionally, evaluations should include both short-term and long-term clinical outcomes, such as the persistence of effects in the postoperative phase and metrics concerning cancer recurrence and progression.

## 10. Conclusions

Ketamine appears highly promising within the oncological context; however, there is a lack of sufficient evidence to support its widespread use in clinical anesthesia practices for cancer patients beyond its existing applications. There is a crucial need for the standardization of clinical research methodologies and an improved assessment of both short-term and long-term clinical results.

## Figures and Tables

**Table 1:** Laboratory studies on anti-inflammatory and immunomodulatory effects of ketamine.

First Author	Model Type	Ketamine Dosing and Route	Other Interventions	Main Results Regarding Inflammation
Taheri et al. (2022) [69]	In vivo, sepsis-like state in mice subjected to middle cerebral artery occlusion	10 mg/kg IP	LPS from *E. coli* 4 mg/kg	Treatment with ketamine significantly decreased IL-1β and IL-10. Ketamine significantly attenuated the sepsis-associated rectal temperature reduction, reduced neurological deficits, reduced infarct volume, and promoted neuronal survival.
Forget et al. (2010) [10]	In vivo, murine model	10 mg/kg IP	Sevoflurane 3–8%.	NK activity varies according to the same time course as control animals. Reduction in lung metastases.
Zhou et al. (2019) [70]	In vitro, T cells of patients with refractory cancer pain	T cells were treated with 100 ng/mL ketamine in combination with morphine	Morphine 200 ng/mL	Decreased CD4+, CD8+ T cells; reduced IL-2, IFN-γ mRNA levels; no significant alteration in IL-2, IFN-γ concentration. No significant exacerbation by low-dose ketamine.
Zhou et al. (2017) [71]	In vitro, T cells of patients with refractory cancer pain	T cells were treated with 100 ng/mL, 500 ng/mL, and 1000 ng/mL ketamine in combination with morphine	Morphine 200 ng/mL	Decreased production of IL-2, IFN-γ; inhibited production of TNF-a, IL-6, IL-8.
Spencer et al. (2023) [72]	In vivo, Murine model	0, 10, or 40 mg/kg, IV infusion for 2 h		Reduced pro-inflammatory cytokines (IL-6, TNF-α); dose-dependent effects. Sex-related differences in the effects on peripheral inflammatory markers in rodents.
Zhao et al. (2023) [73]	In vitro, PC12 cells—rat pheochromocytoma cell line	0.25 μg/mL, in vitro	LPS 10 μg/mL	Reduced neuronal apoptosis; inhibited TLR4/MAPK/NF-κB signaling; decreased IL-6, IL-1β, TNF-α expression.
Melamed et al. (2003) [74]	In vivo, murine model	80 mg/kg IP; 74 mg/kg IV	Halothane 2–3%; diazepam SC 12 mg/kg; nadolol SC, 0.4 mg/kg	Suppressed NK cell activity, increased lung tumor retention and number of metastases. Potential mitigation by β-adrenergic blockers.
Kawasaki et al. (1999) [62]	In vitro, human whole blood	0–500 mg/mL, in vitro	LPS 10 ng/mL, rhTNF-a 104 U/mL	Ketamine inhibits production of proinflammatory cytokines IL-6, IL-8, TNF-α.
Kawasaki et al. (2001) [63]	In vitro, human whole blood	0–1000 µM, in vitro	Staphylococcal enterotoxin B 10 ng/mL	Both S(+)- and R(−)-ketamine suppressed SEB-induced TNF-, IL-6, and IL-8 production.
Meyer et al. (2004) [67]	In vivo, burn injury murine model	90 mg/kg IP	29% body surface third-degree burns	Ketamine reduced mortality rate compared to midazolam and fentanyl.
Shaked et al. (2003) [54]	In vivo, sepsis murine model	50 mg/kg IP	*E. coli* inoculation 0.4 × 10^9^ CFU	Ketamine improved survival rates and suppressed IL-6 and TNFα production.
Taniguchi et al. (2003) [68]	In vivo, Endotoxin-Induced Shock murine model	5 mg/kg/h, 10 mg/kg/h, 20 mg/kg/h IV	*E. coli* endotoxin 10 mg/kg	Ketamine inhibited hypotension, metabolic acidosis, and cytokine responses (IL-6, TNF-α), and improved survival rates.
Gurfinkel et al. (2006) [66]	In vivo, burn injury and sepsis murine model	10 mg/kg IP	30% body surface area full-thickness burns + E. coli inoculation (0.2 × 10^9^ CFU)	Ketamine improved survival rates and decreased IL-6 production.

CFU—Colony Forming Unit; *E. coli*—*Escherichia coli*; IFN-γ—Interferon-γ; IL—Interleukin; IP—Intraperitoneal; IV—Intravenous; LPS—Lipopolysaccharide; MAPK—Mitogen-Activated Protein Kinase; TLR—Toll-Like Receptor; TNF-α—Tumor Necrosis Factor-α; NK—Natural Killer; NF-κB—Nuclear Factor-kappa B; SEB—Staphylococcal Enterotoxin B; SC—Subcutaneous.

**Table 2:** Clinical studies on anti-inflammatory and immunomodulatory effects of ketamine.

First Author	Model Type and Type of Study	Ketamine Dosing and Route	Other Interventions	Effect on Inflammatory or Immune Markers	Conclusions Regarding the Effect of Ketamine
Dale et al. (2012) [14]	Systematic review and meta-analysis; RCTs	Varied dosing: ketamine single dose of 1–2 mg/kg; infusion 1–3.5 mg/kg/h S-ketamine single dose of 0.15–4 mg/kg; infusion 0.075–4 mg/kg/h	No standardization in anesthesia protocol. Some studies used Methylprednisolone, dexamethasone, ibuprofen	Reduction in IL-6, increase in IL-10, effects on CRP, IL-8, TNF-alpha	Significant inhibitory effect on early postoperative IL-6 response; potential anti-inflammatory role.
El Sherif et al. (2022) [80]	Randomized clinical trial	Local wound infiltration with ketamine 2 mg/kg + local anesthetic	Bupivacaine 40 mL of 0.25%; Anesthesia: fentanyl 1 μg/kg, propofol 2 mg/kg, and rocuronium 0.6 mg/kg and maintained with sevoflurane	Decrease in IL-6, TNF-α, increase in IL-10, stable IL-1β levels	Effective attenuation of postoperative inflammatory response; significant pain reduction; reduction in morphine consumption.
Roytblat et al. (1998) [75]	Randomized, double-blind, prospective clinical trial	0.25 mg/kg; bolus during induction of anesthesia	IM injections of morphine 0.1 mg/kg and scopolamine 0.3–0.4 mg, 1–2 mg of midazolam IV. Anesthesia: fentanyl 15 mcg/kg, midazolam 10 mg, and pancuronium 0.1 mg/kg. Maintenance, isoflurane, fentanyl (3–5 mcg/kg) and midazolam (1–2 mg)	Significant suppression of serum IL-6 response post-CPB	Potential anti-inflammatory effect, beneficial in reducing postoperative complications.
Beilin et al. (2007) [76]	Randomized, double-blind clinical trial	0.15 mg/kg; IV before induction of anesthesia	Diazepam 5–10 mg given orally 90 min before operation and with IV administration of midazolam 2–3 mg. Anesthesia: fentanyl 2–3 mg/kg, thiopentone 4–6 mg/kg, and vecuronium 0.1 mg/kg, maintained with nitrous oxide, isoflurane, and the addition of fentanyl.	Attenuation in secretion of the proinflammatory cytokines IL-6 and TNF-a, and in the preservation of IL-2 production at its preoperative level.	Beneficial effect on immune response during early postoperative period.
Zhang et al. (2023) [92]	Prospective, randomized, controlled trial	0.25, 0.5, or 0.75 mg/kg induction dose followed by infusion of 0.25, 0.5, or 0.75 mg/kg/h	Penehyclidine 1 mg orally. Midazolam (0.05 mg/kg), propofol (2.0 mg/kg), and rocuronium (0.8 mg/kg) and maintained with a target-controlled infusion of propofol (4–6 mg/kg/h), remifentanil (0.2–0.6 μg/kg/min)	Increased counts of CD3+ and CD4+ cells, opioid-sparing effects, reduced VAS scores	Anti-inflammatory and immune protective role; dose-related effects.
Alhayyan et al. (2020) [82]	Systematic review and meta-analysis; RCTs	Not specified (analysis of multiple studies)	Use of ketamine compared to either placebo or opiates during GA; not specified	Reduction in CRP, no significant change in IL-6	Moderation of postoperative systemic inflammatory response; the studies were heterogeneous and generally of low quality.
Cho et al. (2021) [23]	Randomized controlled trial	0.25 mg/kg bolus, then 0.05 mg/kg/h; intravenous	Anesthesia: propofol (1.5–2 mg/kg) and remifentanil (1 mcg/kg). Rocuronium (0.6 mg/kg). Anesthesia was maintained with 4–7 volume% desflurane and an intravenous infusion of remifentanil (0.05–0.1 mcg/kg/min). Fentanyl 50 mcg single dose and 0.3 mg ramosetron; 1 mg neostigmine with 0.2 mg glycopyrrolate	No significant change in NK cell activity or IL-6 levels	When added as an adjunct to desflurane anesthesia, subanesthetic low-dose ketamine did not exert beneficial immunomodulatory or inflammatory responses, or improve prognosis in colorectal cancer surgery patients.
Zhan et al. (2020) [81]	Open-label clinical trial	Six infusions of 0.5 mg/kg, IV, over a 12-day period	Concomitant use of antidepressant, mood stabilizers, benzodiazepines, antipsychotics. Dosing not specified	Downregulated: GM-CSF, fractalkine, IFN-γ, IL-10, IL-12p70, IL-17A, IL-1β, IL-2, IL-4, IL-23, IL-5, IL-6, IL-7, TNF-α	Inflammatory cytokines associated with depression severity; alterations correlate with symptom improvement.
Ali et al. (2017) [79]	Randomized controlled trial	Single dose of S-ketamine 0.5 mg/kg, IV, or repeated dose S-ketamine of 0.2 mg/kg, IV, at 20 min intervals	Midazolam 1 mg and ranitidine 50 mg. Propofol (2 mg/kg), fentanyl (1 µg/kg), atracurium (0.5 mg/kg). Epidural levobupivacaine (5 mL 0.25%). Atracurium (8 µg/kg/min) and 1%–1.5% isoflurane	Decreased: TNF-α, IL-6	Significant decrease in pro-inflammatory cytokines, especially when given in repeated doses.
Singh et al. (2020) [78]	Prospective observational study	0.5 mg/kg, IV	Complete anesthesia protocol not specified. Alprazolam 0.25 mg and ranitidine 150 mg orally. Propofol (2 mg/kg).	Decreased: CRP, IL-6 (no significant effect on TNF-α, CK-MB, troponin T)	Anti-inflammatory potential of ketamine in preventing immune function alterations attributable to anesthesia and cardiac surgery.
Ren et al. (2022) [77]	Randomized, double-blind, controlled trial	Single dose of 0.1/0.2/0.3 mg/kg, IV	Midazolam (0.05 mg/kg), propofol (2 mg/kg), sufentanil (0.5 μg/kg), cisatracurium (0.2 mg/kg). 1–3% sevoflurane, propofol (6–8 mg/kg/h), remifentanil (0.1–0.2 μg/kg/min), cisatracurium (5 mg/h), intermittent sufentanil (10–20 μg)	Dose-dependent decrease in IL-6, IL-8, TNF-α	Ketamine (0.3 mg/kg) can significantly improve postoperative anxiety and depression among colorectal cancer patients and reduce anti-inflammatory markers.

CRP—C Reactive Protein; CPB—Cardiopulmonary Bypass; CK-MB—Creatine Kinase-MB; GA—General Anesthesia; GM-CSF—Granulocyte–Macrophage Colony-Stimulating Factor; IFN-γ—Interferon-γ; IL—Interleukin; IM—Intramuscular; IV—Intravenous; NK—Natural Killer; RCT—Randomized Controlled Trial; TNF-α—Tumor Necrosis Factor-α; VAS—Visual Analogue Scale.

**Table 3:** Laboratory studies of the direct effect of NMDA antagonism on cancer cell biology.

First Author	Model Type	Ketamine Dosing and Route	Conclusions Regarding Ketamine’s Effect	Specifics of Cancer Cell Biology
Malsy et al. (2015) [35]	In vitro study on human pancreatic adenocarcinoma cell lines	Cell proliferation analysis: Cells were incubated for 48 h (1000 μM ketamine, s-ketamine, and MK 801). Apoptosis analysis: PaTu8988t and Panc-1 cells were incubated (10 μM ketamine, s-ketamine, MK 801) for up to 24 h	Inhibited cell proliferation and decreased apoptosis rate in pancreatic cancer cells	Expression of NMDA receptor type R2a in cells; noncompetitive blockade of the NMDA receptor complex
He et al. (2021) [102]	In vitro and in vivo study on liver cancer cell lines and murine model	In vitro: ketamine 10 μg/mL up to 72 h; In vivo: ketamine 20 mg/kg, intraperitoneally	Suppressed the viability and proliferation of liver cancer cells; induced ferroptosis	Ketamine downregulates GPX4 via the lncRNA PVT1/miR-214-3p pathway
Zhou et al. (2018) [36]	In vitro study on A549 cell line derived from lung adenocarcinoma	Ketamine concentrations of 0, 1, 10, and 100 μmol/L for 24 h	Induced apoptosis in A549 cells in a concentration-dependent manner through the upregulation of CD69 expression	Upregulation of CD69 expression; potential therapeutic target
Hu et al. (2020) [100]	In vitro and in vivo study on colorectal cancer cell lines and murine model	In vitro: incubation with 1, 5, and 10 μg/mL ketamine for 4 h; In vivo: CT26 cells in BALB/c mice were treated with subcutaneous ketamine (5 mg/mL) for 4 h	Inhibited aerobic glycolysis in colorectal cancer cells; tumor volumes were reduced.	Disruption of NMDA receptor-CaMK II-c-Myc pathway; impact on cell survival and proliferation
Duan et al. (2019) [37]	In vitro study on human colorectal cancer cells	Varying concentrations of ketamine (1, 5, 10 μg/mL) for 4 h	Ketamine treatment inhibited colon cancer cell viability and migration in HT29 and SW480 cells in a concentration-dependent manner.	Inhibited malignant potential of colorectal cancer cells. Decreased aerobic glycolysis and decreased the expression of glycolysis-related proteins in HT29 and SW480 cells. Ketamine inhibited c-Myc expression and CaMK II phosphorylation and decreased calcium levels.
Chen et al. (2022) [103]	In vitro and in vivo study on breast cancer cell line EO771 and addiction murine model	In vitro: ketamine 0, 1, 10, and 100 µM for 7 days; In vivo: 30 mg/kg intraperitoneally, daily, for 14 days	Enhanced migration and invasion of EO771 cells. Increased breast tumor volume and weight in ketamine-addicted mice	Upregulation of miR-27b-3p, HER2, and EGFR in breast tumors
He et al. (2013) [104]	In vitro experiments on human breast cancer cell line MDA-MB-231	Ketamine (1, 10, and 100 µM) for 24 h	Upregulates the expression of the anti-apoptotic protein Bcl-2	Potential negative side effect of ketamine in the context of breast cancer growth
Malsy et al. (2019) [105]	In vitro analysis on pancreatic carcinoma cell line PaTu8988t	Ketamine 5 μM, S-ketamine 5 μM for 0, 24, 48, and 72 h.	Cell proliferation was significantly decreased after 48 h stimulation with ketamine or s-ketamine	Inhibition of NFATc2 transcription factor in pancreatic carcinoma

CaMK II— Ca^2+^/calmodulin-dependent protein kinase II; EGFR—Epidermal Growth Factor Receptor; GPX4—Glutathione Peroxidase 4; HER2—Human Epidermal Growth Factor Receptor 2; NMDA—N-Methyl-D-aspartate.
